# N-terminal truncating variants in *CACNB1* cause a new congenital muscular disorder

**DOI:** 10.1038/s41431-025-01944-4

**Published:** 2025-09-29

**Authors:** Asier Iturrate, Nurit Assia Batzir, Ranit Jaron, David Garcia-Valentin, Julian Nevado, Jair Tenorio-Castano, Pablo Lapunzina, Kamila Lee, Rotem Greenberg, Dvora Sassi, Sharon Aharoni, Alla Kuzminsky, Lina Basel-Salmon, Naama Orenstein, Yakov Fellig, Shay Ben-Shachar, Dina Marek-Yagel, Victor L. Ruiz-Perez

**Affiliations:** 1 Instituto de Investigaciones Biomédicas Sols-Morreale (IIBM), Consejo Superior de Investigaciones Científicas-Universidad Autónoma de Madrid, Madrid, Spain; 2 CIBER de Enfermedades Raras, Instituto de Salud Carlos III, Madrid, Spain; 3 Pediatric Genetics Unit, Schneider Children’s Medical Center of Israel, Petah Tikva, Israel; 4 Instituto de Genética Médica y Molecular (INGEMM), Hospital Universitario La Paz-IdiPAZ, ITHACA-ERN, Madrid, Spain; 5 Clalit Research Institute, Clalit Health Services, Ramat Gan, Israel; 6 Clalit Genomic center, Clalit Health Services, Petach Tikva, Israel; 7 Gray Faculty of Medical and Health Sciences, Tel Aviv University, Tel Aviv, Israel; 8 Institute of Pediatric Neurology, Schneider Children’s Medical Center of Israel, Petah Tikva, Israel; 9 Raphael Recanati Genetic Institute, Rabin Medical Center-Beilinson Hospital, Petah-Tikva, Israel; 10 Felsenstein Medical Research Center, Rabin Medical Center, Petah-Tikva, Israel; 11 Department of Pathology, Hadassah Medical Center and the Faculty of Medicine, The Hebrew University, Jerusalem, Israel; 12 The Ivan and Francesca Berkowitz Family Living Laboratory Collaboration, at Harvard Medical School and Clalit Research Institute, Boston, MA USA

**Keywords:** Neuromuscular disease, Disease genetics

## Abstract

Excitation-contraction (EC) coupling is an essential process for skeletal muscle function. Pathogenic variants in different EC coupling components have previously been associated with various neuromuscular disorders. In this study we aimed to identify the genetic etiology of a muscular condition characterized by early-onset muscle weakness, elevated CK, ptosis and low body weight, which was observed in three individuals from two unrelated consanguineous families. Exome sequencing (ES) performed in multiple individuals of one family, and ES in combination with SNP array-based homozygosity mapping in the proband of the other family, revealed different homozygous loss-of-function variants in the second exon of *CACNB1* in the affected individuals from each family. *CACNB1* encodes the β1 subunit of the skeletal muscle dihydropyridine receptor (DHPR), a voltage-gated Ca^2+^ channel with a major role in EC coupling. Molecular impact of the identified variants was assessed in LHCN-M2 human myoblasts. Long-read RNA sequencing in LHCN-M2 wild-type myotubes showed that in differentiated skeletal muscle cells virtually all *CACNB1* transcript isoforms contain exon 2 and will therefore be affected by genetic variants in this exon. Pathogenicity of the identified *CACNB1* variants was further validated by replicating one of them (c.85-1G>A) in LHCN-M2 cells using CRISPR-Cas9-mediated base-editing. Analysis of LHCN-M2 edited myotubes demonstrated that in addition to the loss of β1 subunits, these cells displayed severely reduced protein levels of α1S, the pore-forming subunit of DHPR. We conclude that pathogenic variants in *CACNB1* cause a new congenital muscular disorder.

## Introduction

Voltage-gated calcium channels (VGCCs) are multi-protein transmembrane complexes that function as electrical signal transducers in vital physiological processes including skeletal and cardiac muscle contraction, neuronal synaptic transmission, hormone release, and Ca^+2^-mediated regulation of gene expression. There are several categories of VGCCs, namely L-, N-, P/Q-, R- and T-type which differ in their voltage-dependence, kinetics and sensitivities to pharmacological compounds [1, 2]. In particular, the L-type calcium channels, also named Cav1, are characterized by slow voltage-dependent inactivation and susceptibility to calcium antagonist drugs, such as dihydropyridines and phenylalkylamines [1, 3, 4].

The process of excitation-contraction (EC) coupling in skeletal muscle largely depends on the L-type calcium channel Cav1.1 or dihydropyridine receptor (DHPR) [1, 5]. This channel localizes to T-tubules which are invaginations of the membrane of myofibers positioned next to terminal cisternae of the sarcoplasmic reticulum (SR). EC coupling initially involves the depolarization of the myofiber cell membrane at the neuromuscular junction secondary to an impulse from a nerve fiber. Electrical changes then spread along the sarcolemma inducing DHPRs to undergo a conformational change that is mechanically transmitted to Ca^2+^ release channels of the terminal SR, the ryanodine receptors (RYR1), causing their aperture. As a result, Ca^2+^ ions stored in the SR are released into the cytosol, subsequently leading to muscle contraction [5]. In addition, DHPRs allow the entry of extracellular Ca^2+^ into the cell in response to the depolarization of the cell membrane [6, 7].

VGCCs comprise a pore-forming α1 subunit and three auxiliary proteins, including a cytoplasmic β subunit, an extracellular α2/δ subunit, and a transmembrane ϒ subunit [1]. These proteins are encoded by ten *CACNA1* (α1), four *CACNB* (β), four *CACNA2D* (α2/δ) and eight *CACNG* genes (ϒ) [2, 8]. The diversity of VGCC channels is further incremented through alternative splicing of the VGCC subunits [9, 10]. Specifically, the DHPR of skeletal muscle results from the association of the muscle-specific α1S (Cav1.1) subunit with accessory proteins β1, α2/δ-1 and γ1 [11].

*CACNB1* is the gene coding for the auxiliary subunit β1. It has long been established that this gene gives rise to three different protein isoforms, β1a, β1b and β1c, through alternative splicing of exons 7 and 13. The β1a isoform, which incorporates exons 7a and 13a, is expressed in skeletal muscle, and is traditionally recognized as the major *CACNB1* transcript in this tissue, being actually regarded as the β isoform of the DHPR of skeletal muscle. Although less significantly, β1a is also expressed in the heart [12–14]. Splice variants β1b/β1c had a different expression pattern and were detected in brain, heart and spleen [13, 14]. In addition to the β1a,b,c mRNAs, a new *CACNB1* transcript isoform having a different alternative first exon was recently reported in skeletal muscle [15].

At a structural level, all β subunits share a central part comprising two regions of conserved amino acids, a Src homology 3 (SH3)-domain and a guanylate kinase (GK)-domain, joined by a HOOK-domain [16, 17]. The β subunits interact with a cytoplasmic segment of the α1 subunits [18] and have been suggested to promote α1 membrane trafficking acting as α1 chaperones, and/or covering an ER retention signal on α1, or preventing α1 ubiquitination [19]. The kinetics and voltage-sensing characteristics of the α1 channels were also shown to be influenced by the β subunits [20].

Disruption of EC coupling is a major pathomechanism underlying muscular disease [21]. Pathogenic variants in the α1S-encoding gene, *CACNA1S*, result in various muscular disorders including congenital myopathy (CM; MIM: 620246), hypokalemic periodic paralysis (MIM: 170400) and malignant hyperthermia susceptibility (MIM: 601887) [11, 22]. Similarly, *RYR1* variants also cause several types of muscular conditions [22]. However, no human genetic disorder associated with the β subunit of the skeletal muscle DHPR has been recognized until now. Herein we describe the identification of homozygous loss-of-function variants in *CACNB1* in three patients with muscle weakness, elevated creatine kinase (CK) and low body weight.

## Materials and methods

Materials and Methods of this article are provided as supplementary information.

## Results

### Clinical presentations

Probands include two cousins from a large, highly consanguineous, Bedouin family (family 1) who presented with muscle wasting and weakness and a third patient from an unrelated consanguineous family (family 2) of Egyptian origin with a similar condition.

**Fig. 1:**
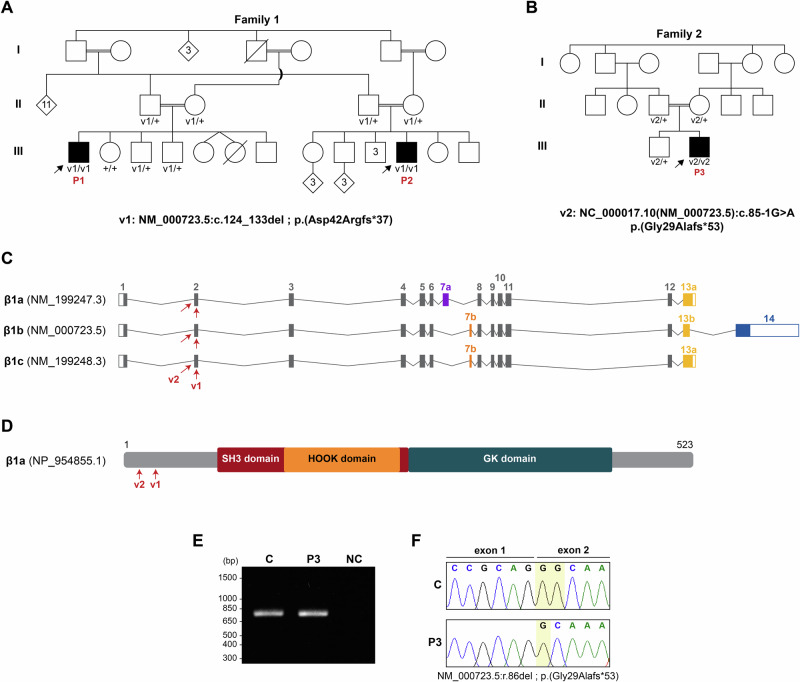
Identification of homozygous pathogenic variants in *CACNB1* in patients with congenital muscular disease. Pedigrees of patients from family 1 (**A**) and family 2 (**B**). Probands (P1, P2 and P3) are indicated with arrows. Genotype of individuals molecularly tested is designated underneath. +: wild-type allele; v1: variant of family 1; v2: variant of family 2. **C** Schematic diagram showing the position of variants v1 and v2 (arrows) in *CACNB1* transcript isoforms (β1a-c). Alternatively spliced exons 7 and 13 are colored. 5´and 3´UTRs are represented as empty boxes. **D** Schematic representation of β1a protein illustrating conserved SH3 and GK domains and the HOOK domain. Variants v1 and v2 are depicted. **E** Representative agarose gel image showing the result of an RT-PCR assay between exons 1 and 9 of *CACNB1* in whole blood RNA from proband P3 and a control sample (C), *n* = 3. NC: non-template control. **F** Sanger sequencing chromatograms of the RT-PCR products shown in (**E**) demonstrating the deletion of the first nucleotide of *CACNB1* exon 2 in the RT-PCR product of P3. Nucleotides highlighted in yellow illustrate the deletion of a G.

The first proband of family 1 (P1; Fig. 1A; Table 1) is the first of seven siblings, born to first-degree cousins. No amniocentesis or echocardiogram was performed during pregnancy. He was born via spontaneous delivery at full term weighing 2600 g. Hypotonia was noted since birth and he began walking at the age of 3 years. The patient was referred for genetic evaluation due to muscle weakness and elevated CK levels (972 and 1992 U/L measured at age 15 years; reference values 20–200 U/L). EMG findings suggested myopathy without evidence of neuropathy. Cardiological evaluation at 16 years of age revealed non-compaction cardiomyopathy with mild enlargement of the left ventricle. Pulmonary evaluation at age 18 years showed a restrictive pattern on spirometry with forced vital capacity at 47%, likely due to respiratory muscle weakness as well as restriction caused by chest wall deformities. Chest X-ray showed no lobar opacities or atelectasis.

**Table 1:** Clinical features of patients with homozygous pathogenic variants in *CACNB1*.

	P1	P2	P3
*CACNB1* variant	c.124_133del	c.124_133del	c.85-1G>A
Demographic information			
Sex	Male	Male	Male
Age at onset (clinical findings)	At birth (undescended testes, borderline LBW, hypotonia)	At birth (undescended testes, LBW, hypotonia - noted at age 4 months)	At birth (undescended testes, sub-mucous cleft palate, congenital bilateral ptosis, LBW)
Age at last assessment	19 years	17 years	15 years
Neuromuscular			
Hypotonia	+	+	−
Muscle weakness			
Axial	No head drop	No head drop	No head drop
Facial	Myopathic facies, ptosis (mild)	Myopathic facies, ptosis (severe), ophthalmoplegia	Myopathic facies, ptosis (severe)
Proximal	+	++ (LL > UL)	+
Distal	−	+ (mild)	−
Muscle wasting	++	++	+
Chest wall deformity	+	+	+
Hand/foot abnormalities	Slightly high arch of feet, hyperlaxity of fingers	Slightly high arch of feet, thenar and hypothenar wasting	−
Contractures	−	−	−
Decreased/absent DTRs	+	+	+
Development			
Motor delay (age of first walking)	+ (3 years)	+ (3.5 years)	+ (1.5 years)
Speech delay (age of first words)	− (1 year)	+ (2.5 years)	− (1.5 years)
Cardiac			
Cardiomyopathy	LVNC	−	−
Pulmonary			
FVC	47%	NA	NA
Pattern	Restrictive	NA	NA
Craniofacial			
Dolichocephaly	+	+	−
Palate abnormalities	High, narrow palate	High, narrow palate	Sub-mucous cleft palate
Dental crowding	NA	NA	+
Growth			
Birth weight (gestational age)	2600 g (full term)	2100 g (full term)	1850 g (full term)
Failure to thrive	+	+	−
Lab and imaging			
CK			
Infancy	Normal	Normal	NA
Adolescence	1000–2000 U/L (reference range: 20–200 U/L)	700–5000 U/L (reference range: 20–200 U/L)	2900–7300 U/L^a^ (reference range: 26–192 U/L)
EMG/NCS:			
Myopathy	+	+	+
Neuropathy	−	Mild peroneal neuropathy	−
MRI	NA	NA	fibroadipose replacement

*LBW* low birth weight, *LL* lower limbs, *UL* upper limbs, *DTRs* deep tendon reflexes, *LVNC* left ventricular noncompaction cardiomyopathy, *FVC* forced vital capacity, *NA* not assessed, *CK* creatine kinase, *EMG/NCS* electromyography/nerve conduction studies, *MRI* Magnetic resonance imaging.

^a^Between 9 and 13 years of age.

On the last evaluation at age 19 years, head circumference was normal (54.5 cm, 34th percentile), height was 163 cm (−1.83 SD), and weight was 32.8 kg (−6.8 SD). He exhibited a myopathic face, dolichocephaly, a narrow palate, and a wide uvula. Bilateral pectoralis muscle atrophy, pectus excavatum with asymmetric, narrow chest wall, hypotonia and decreased deep tendon reflexes were noticed. He demonstrated the ability to stand and walk on toes and heels, with proximal weakness in all four extremities. Winged scapula and hyperextension in the hands and palms were also observed. Intelligence was normal.

Proband 2 (P2; Fig. 1A; Table 1) is the 6th child out of eight born to first cousin parents. He is the paternal and maternal cousin of P1 (Fig. 1A). He was born at full term following a normal pregnancy, no polyhydramnios was reported. Birth weight was 2100 g. He was followed up since infancy for hypotonia, muscle weakness, failure to thrive and ptosis. He walked at age 3 years and a half and talked at two years and a half. The patient was seen by neuromuscular specialists and was noted to have muscle wasting and proximal weakness. He ambulates using a wheelchair since age 14 years.

CK was normal in the first year of life but was found to be increased to ~800 U/L (reference values 20–200 U/L) in adolescence. EMG showed moderate non-irritable myopathy without evidence of polyneuropathy or myotonia. Mild neuropathy of the left peroneal nerve was seen. An echocardiogram at age 16 years was normal, and a chest X-ray revealed scoliosis and deformity of the rib cage. Pulmonary function was not evaluated. A muscle biopsy at 22 months of age showed mild non-specific findings that included variations in myofiber diameter without increase in central nuclei, necrosis or regenerating fibers. Sparse atrophic muscle fibers were also observed (Supplementary Fig. 1).

On last exam, at age 17 years, head circumference was at the 34th percentile, his height was 163 cm (5th percentile) and weight was 36 kg (−5.18 SD). He had slight dolichocephaly, a long myopathic face, ptosis and limitation of upward gaze and a tented mouth with a high, narrow palate. Assessment of muscle strength showed proximal muscle weakness in all four limbs as well as some distal weakness. Severe muscle wasting was noted in torso, upper and lower limbs including atrophy of the thenar and hypothenar muscles in the hands. There were no muscle contractures. He also had kyphosis and pectus excavatum. He had some learning difficulties but intelligence was reported as normal.

The siblings of P1 and P2 do not show signs and symptoms of muscular disease. Of note, a sister of P1 died at age 2.5 years from complications of a congenital cardiac malformation; no other information on her condition was available. Two siblings of P1 were diagnosed with Usher syndrome, with P1 being heterozygous for the family variant (NM_032119.4(*ADGRV1*):c.12125del (p.Met4042fs); ClinVar: pathogenic). One sibling of P2 had epilepsy.

Proband 3 (P3; Fig. 1B; Table 1) is the second child born to healthy, consanguineous parents. He was delivered by cesarean section at full term (39 gestational weeks) after an uneventful pregnancy. Growth parameters at birth were normal (10–25th percentile) for length and occipitofrontal circumference, but below average for weight (1850 g, <3rd percentile). Submucous cleft palate, congenital bilateral ptosis and undescended testicles were all documented at birth, for which he underwent surgical correction. During early childhood, the patient showed normal growth and development.

The patient first presented for genetic assessment at age 9 years complaining of muscle weakness and frequent falling. EMG performed at the age of 12 years indicated myopathic changes affecting proximal muscles, and elevated CK levels were identified on three occasions between 9–13 years of age (2900, 6500, and 7300 U/L; reference range 26–192 U/L). On this basis the patient was diagnosed with a congenital muscle disease. MRI of pelvic and thigh muscles at age 12 years revealed partial fibroadipose replacement targeting multiple groups of muscles bilaterally and symmetrically, with the glutei maximus followed by the adductor magnus and vastus lateralis being the most affected muscles. The MRI study also revealed normal size and shape as well as signal characteristics of extraocular muscles bilaterally. The overall MRI features also matched with an underlying myopathy. The patient did not exhibit facial dysmorphology apart from a striking bilateral ptosis and dental overcrowding. He had mild pectus excavatum. There were no relevant complaints that required the assessment of respiratory functions. Conventional cytogenetic studies revealed normal male karyotype, and brain MRI and echocardiogram conducted at 15 years old were normal. On last examination at age 15 years, he showed a stable clinical course, the muscle weakness did not deteriorate, and he had reached puberty around age 14 years. He has also shown good academic achievements.

### Identification of pathogenic variants in *CACNB1* as a new cause of inherited muscular disease

Molecular testing using exome-based myopathy and muscular dystrophy panels was conducted for affected individuals of family 1 (P1 and P2), with no positive findings. ES was subsequently performed in the two affected individuals of this family and seven healthy first-degree relatives (Fig. 1A). Advanced analysis for shared variants between the affected individuals revealed a homozygous 10 bp deletion in *CACNB1* (NM_000723.5:c.124_133del; p.(Asp42Argfs*37); NC_000017.10:g.37351177_37351186del). This variant is located in exon 2 of *CACNB1* and results in N-terminal truncation of β1a-c protein isoforms (Fig. 1C, D). The variant was found to segregate with the disease in the family with only the two affected individuals having the 10-nucleotide deletion in a homozygous state (Fig. 1A and Supplementary Fig. 2A, B). No rare variants of likely clinical relevance in established disease-causing genes known to be associated with the phenotypes were detected. The c.124_133del *CACNB1* variant was absent from an in-house database containing more than 2000 exomes of individuals with variable phenotypes, as well as from public databases (gnomAD v4.0, https://gnomad.broadinstitute.org). Following the American College of Medical Genetics and Genomics (ACMG) and ClinGen guidelines, the variant was classified as PM2_supporting (absent from controls), PM3_supporting (variants in trans), PP1_moderate (segregation with the phenotype) [23]. Proband 2 was also found to be homozygous for a pathogenic/likely pathogenic variant in *MVK* (NM_000431.4:c.1129G>A; p.(Val377Ile)) reported in ClinVar. While this variant may contribute to his failure to thrive and learning difficulties, it was considered that it did not explain his severe muscle disorder. The *MVK* variant has an allele frequency of 0.2097% in gnomAD v4.1.

ES was also conducted in the proband of family 2 (P3; Fig. 1B). Considering that the parents of this patient were unaffected and related as first-cousins, the condition was assumed to be recessive and the causative variant homozygous by descent. On this basis, DNA of the patient was hybridized against whole-genome SNP-arrays to identify chromosomal regions of homozygosity (ROHs) susceptible to containing the mutated gene. The results of ES were combined with that of the SNP-arrays, and we prioritized rare coding and splicing pathogenic variants (MAF ≤ 0.01 in gnomAD), that were additionally located within ROHs of the patient larger than 1.5 Mb. This analysis identified a homozygous variant disrupting the canonical acceptor splice site of exon 2 of *CACNB1* (NM_000723.5:c.85-1G>A; NC_000017.10:g.37351224C>T) as the most likely causative variant. This variant was embedded in a 19.68 Mb block of homozygosity and was not listed in gnomAD v4.1.0. Using Sanger sequencing the patient was verified to be homozygous for the c.85-1G>A nucleotide transition, while both parents and the unaffected brother were identified with the variant in the heterozygous state (Fig. 1B and Supplementary Fig. 2C). The *CACNB1* variant of P3 was classified as PM2 and PM3 according to the ACMG criteria.

Bioinformatic evaluation of the c.85-1G>A substitution with the SpliceAI-lookup tool (https://spliceailookup.broadinstitute.org/) indicated that this change eliminates the canonical acceptor splice site of *CACNB1* exon 2 (CAG>CAA) and simultaneously creates another adjacent one (AAG) comprising the first nucleotide of this exon (Supplementary Fig. 3). To determine the impact of the c.85-1G>A variant on *CACNB1* splicing in human tissue, we isolated RNA from a blood sample of P3 and performed RT-PCR between exons 1 and 9 of *CACNB1*. A mix of whole blood RNA from healthy donors was used as control. No differences in the size of the amplified products were observed between patient and control samples. However, in agreement with the bioinformatic prediction, the first nucleotide of *CACNB1* exon 2 was found to be absent from the RT-PCR fragment of the patient after sequencing (Fig. 1E, F). Exclusion of this nucleotide from the *CACNB1* mRNA results in a frameshift at codon 29 which generates a premature termination codon 53 amino acids downstream in the new reading frame, hence also leading to N-terminally truncated β1a-c isoforms [NM_000723.5:r.86del; p.(Gly29Alafs*53)] (Fig. 1C, D).

### Loss-of-function variants in *CACNB1* exon 2 diminish the expression levels of the skeletal muscle DHPR

*CACNB1* is known to undergo tissue-specific alternative splicing. It is well established that this gene gives rise to three different splicing isoforms, β1a, β1b and β1c, all of which share the same amino-terminal region comprising exons 1 and 2 (ref. [13]). The presence of these two N-terminal exons is conserved throughout evolution, and they can be found in distantly related species as molluscan, as well as in the human *CACNB2* and *CACNB4* genes [17]. Residues contained in exon 2 are also evolutionary conserved [17]. Recently, a different β1 transcript initiating in an exon downstream of exon 2 (from now on exon 1b) was reported to be additionally expressed in skeletal muscle tissue of mice and human [15]. Transcript isoforms with a similar alternative 5´exon structure have been described for *CACNB2*, *CACNB4* and the molluscan *CACNB* gene [17]. Taking this into account, we proceeded to evaluate the extent of the pathogenicity of the *CACNB1* variants identified in this study. For this purpose, due to unavailability of biological material from patients, we used the human myoblast cell line LHCN-M2, which can be differentiated into myotubes.

**Fig. 2:**
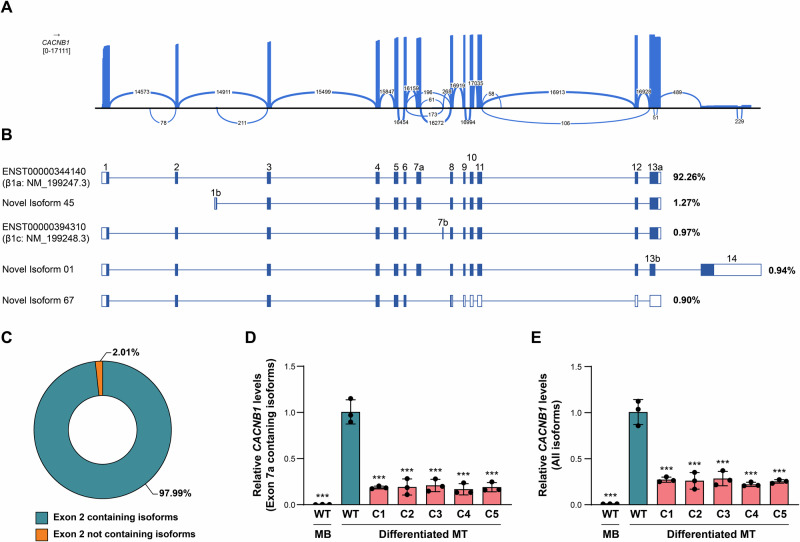
The vast majority of β1 transcripts of LHCN-M2 myotubes include exon 2. **A** IGV sashimi plot from a long-read RNA sequencing experiment in LHCN-M2 myotubes illustrating total reads aligned to the *CACNB1* gene. Exon junctions supported by at least 50 reads are shown. **B** Top 5 most abundant *CACNB1* transcript isoforms in LHCN-M2 myotubes observed through long-read RNA sequencing quantification with IsoQuant. Two mRNA isoforms correspond to annotated transcripts in Ensembl (release 82) and are designated with their transcript IDs, and three are novel transcript isoforms identified by Iso-Seq. Non-coding regions are depicted as empty boxes. Read percentages aligning to each isoform are shown on the right. **C** Pie chart showing the percentage of reads aligned to *CACNB1* transcript isoforms either containing or non-containing the canonical exon 2. **D**, **E** Relative quantification of *CACNB1* mRNA levels in LHCN-M2 myoblasts (MB) and differentiated myotubes (MT) by RT-qPCR using TaqMan gene expression assays targeting isoforms containing the muscle-specific exon 7a (**D**; Hs01120681_m1), or targeting all *CACNB1* isoforms (**E**; Hs 00609497_m1). Wild-type: WT; c.85-1G>A edited clones: C1–C5. Data are expressed as mean ± SD (*n* = 3). ***p < 0.001. One-way ANOVA with Dunnett’s multiple comparisons test relative to the WT-MT control sample. In both expression assays, *CACNB1* expression levels were normalized to the geometric mean of *GAPDH* and *TBP* values. The ΔCt mean value of WT differentiated myotubes was used as the calibrator sample.

To learn about the exon composition of the different *CACNB1* transcript isoforms present in skeletal muscle cells and their respective abundance, we conducted whole transcriptome long-read RNA sequencing of LHCN-M2 differentiated myotubes. Iso-Seq analysis identified 90 novel *CACNB1* transcript isoforms and 5 mRNA variants annotated in Ensembl (release 82), which makes it a total of 95 different *CACNB1* transcripts (Supplementary data 1). Isoform quantification with IsoQuant recognized β1a as the main *CACNB1* mRNA isoform in LHCN-M2 myotubes. This analysis revealed that a 92.26% of the total reads aligning to *CACNB1* transcript isoforms corresponded to the β1a mRNA (Fig. 2A, B, Supplementary Fig. 4 and Supplementary data 2). Notably, only 2.01% of total *CACNB1* reads were assigned to transcripts that did not include exon 2 (Fig. 2C, Supplementary Fig. 4 and Supplementary data 2). Among the mRNA isoforms lacking exon 2, a transcript variant with the same exon 3–13 structure as β1a, but initiating in exon 1b, was the most prevalent (1.27% of total reads) (Fig. 2A, B and Supplementary Fig. 4 and Supplementary data 2). According to this experiment, *CACNB1* frameshift variants occurring in exon 2 affect nearly all *CACNB1* transcripts of differentiated myotubes.

Next, we developed a cellular model for the c.85-1G>A variant by editing the genome of LHCN-M2 myoblasts. This was accomplished by the use of a CRISPR-Cas9-guided cytosine base editor (CBE) system in combination with a sgRNA targeting the acceptor splice site of exon 2. A V106W amino acid change was introduced into the CBE protein since it was previously reported that this helps to adjust the editing window and reduce the number of undesired cytosine modifications [24]. Five correctly edited clonal cell lines, verified to be homozygous for the c.85-1G>A variant by Sanger sequencing, were expanded for further studies (Supplementary Fig. 5A). RT-PCR between exons 1 to 7a in myotubes from the 5 selected clones followed by sequencing of the amplified fragments confirmed that in skeletal muscle cells the c.85-1G>A variant leads to the same splicing defect observed in peripheral blood of P3 (r.86del; p.(Gly29Alafs*53)) (Supplementary Fig. 5B, C).

**Fig. 3:**
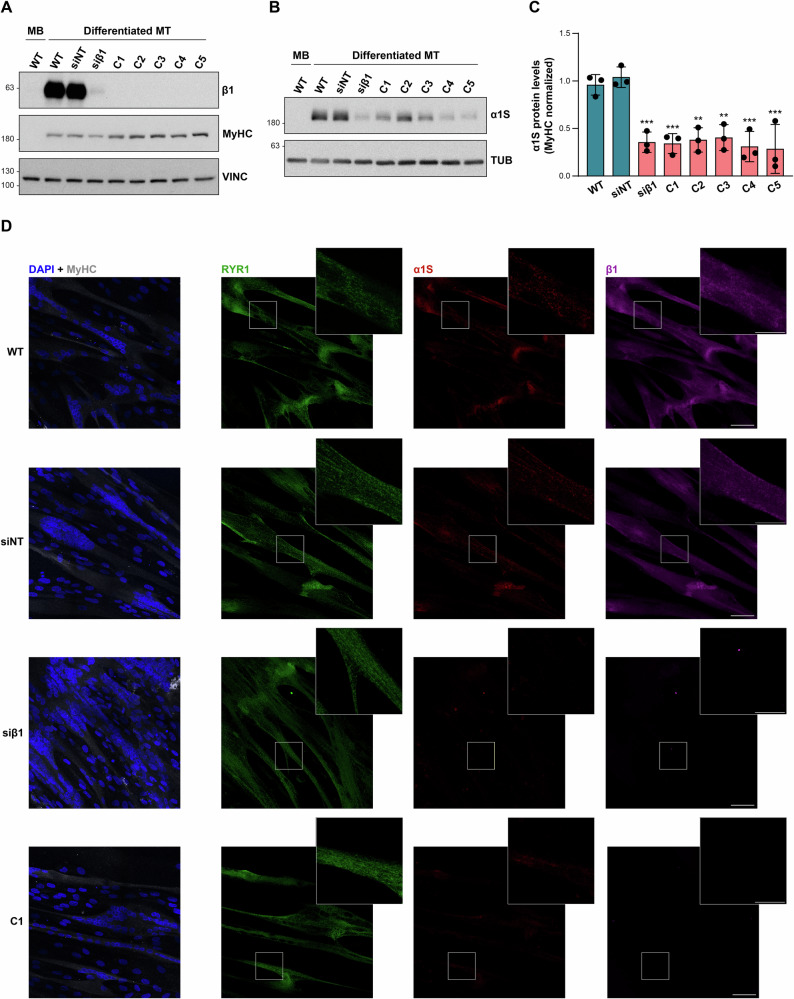
Truncating variants in exon 2 of *CACNB1* impair expression of the α1s subunit of DHPR. **A** Representative immunoblot of β1 in control (WT) and c.85-1G>A edited (C1–C5) LHCN-M2 differentiated myotubes (MT). LHCN-M2 myoblasts (MB), as well as myotubes treated with siRNA against *CACNB1* (siβ1), or with a non-targeted siRNA (siNT), were also included in this experiment. The anti-β1 antibody used was raised against N-terminal residues located in exons 1-2. Protein levels of MyHC served as differentiation maker and vinculin (VINC) acted as loading control. *n* = 3. **B** Immunoblot analysis of α1S in the same protein extracts analyzed in A. Tubulin (TUB) was used as loading control. *n* = 3. **C** Densitometric quantification of α1S levels normalized by the levels of the differentiation marker MyHC. Values are calculated as described in material and methods. Data are expressed as mean ± SD (*n* = 3). **p < 0.01, ***p < 0.001. One-way ANOVA with Dunnett’s multiple comparisons test relative to the WT-MT control sample. **D** Maximum intensity Z-projection of confocal immunofluorescence images of control (WT), siNT-treated, siβ1-treated and c.85-1G>A edited (C1) LHCN-M2 myotubes stained for MyHC, RYR1, α1S and β1. *n* = 3. Nuclei stained in blue with DAPI are shown in the MyHC images. Boxed areas are digitally magnified in inset images. Scale bars: 50 µm (overview images), and 20 µm (magnified insets).

As the expression of β1 and α1S subunits of the DHPR is induced by myogenic differentiation, functional studies in LHCN-M2 *CACNB1*-edited clones were carried out in differentiated myotubes. *CACNB1* expression in these cells was tested through RT-qPCR using two TaqMan expression assays, one targeting *CACNB1* isoforms containing the muscle-specific exon 7a, and another one spanning the constitutive exons 11–12. Both assays demonstrated a severe drop in the amount of *CACNB1* mRNA in the myotubes derived from the mutated clones with respect to the parental LHCN-M2 myotubes, strongly suggesting the activation of the nonsense-mediated mRNA decay (NMD) pathway by the c.85-1G>A *CACNB1* variant (Fig. 2D, E). Immunoblotting with an anti-β1-specific antibody raised against N-terminal exon 1-2 amino acids, also demonstrated absence of β1 subunits comprising these exons in the edited myotubes (Fig. 3A).

Previous studies performed in *Cacnb1*-knockout mice and zebrafish mutants showed that β1 regulates the membrane trafficking of the α1S subunit [25, 26]. Therefore, we compared the expression of α1S both, at the mRNA and protein levels, between *CACNB1*-edited and parental LHCN-M2 myotubes. No statistically significant differences were detected in the amount of α1S mRNA between the two types of cells (Supplementary Fig. 6). However, immunoblot analysis showed the levels of the α1S protein in the c.85-1G>A myotubes to be reduced by 60–71% with respect to the control cultures (Fig. 3B, C). This reduction in the expression of α1S in the myotubes derived from the edited clones was additionally corroborated through confocal immunofluorescence (Fig. 3D and Supplementary Fig. 7).

LHCN-M2 myotubes treated with *CACNB1*-siRNA against all *CACNB1* transcript isoforms were also analyzed for β1 and α1S expression. While myotubes transfected with a non-targeted control siRNA showed normal levels of these proteins, treatment of myotubes with *CACNB1*-siRNA had an effect on the levels of α1S protein similar to that observed in the c.85-1G>A edited clones (Fig. 3A–D). No significant changes in the amount of *CACNA1S* mRNA were observed in these cells after *CACNB1*-siRNA treatment (Supplementary Fig. 6). Hence, both, the downregulation of all *CACNB1* transcript isoforms, and the disruption of only those *CACNB1* transcripts containing exon 2, have a comparable effect on the expression levels of α1S protein. This is consistent with the results obtained through long-read RNA sequencing according to which the exon 2-containing β1a isoform is the most abundant *CACNB1* transcript isoform in differentiated myotubes.

## Discussion

**Fig. 4:**
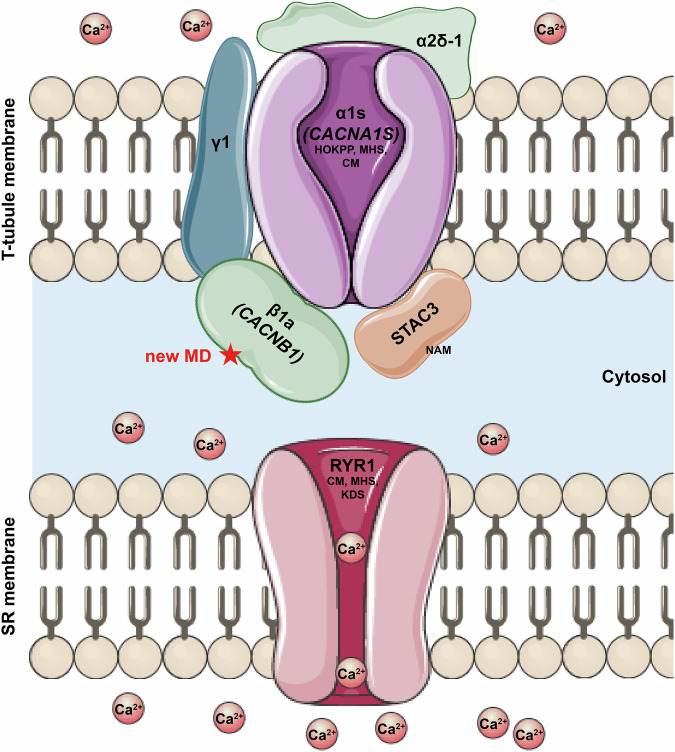
EC coupling associated diseases. Schematic representation of the different subunits of the skeletal muscle DHPR, including the pore-forming subunit α1S (*CACNA1S*), β1 (*CACNB1*), α2/δ-1, and γ1 subunits. The interaction with RYR1 and additional proteins involved in EC coupling such as STAC3 is indicated. Calcium ions are depicted as Ca²⁺. Pathogenic variants in genes encoding EC coupling components are associated with a group of muscle disorders, including hypokalemic periodic paralysis (HOKPP), malignant hyperthermia susceptibility (MHS), congenital myopathy (CM), King-Denborough syndrome (KDS) and Native American myopathy (NAM). The new congenital muscular disease (new MD) associated with *CACNB1* is highlighted in red. Images provided and adapted from Servier Medical Art (https://smart.servier.com/), licensed under CC BY 4.0 (https://creativecommons.org/licenses/by/4.0/).

Dysregulation of EC coupling, which in skeletal muscle relies on the interaction between the DHPR and RYR1, is widely recognized to be a cause of muscular disease. Pathogenic variants in key components of EC coupling such as *CACNA1S*, *RYR1* and *STAC3* are associated with CM as well as other pathologies involving muscle weakness [27–29]. Herein, we report that homozygous null variants in *CACNB1*, encoding the β1 subunit of skeletal muscle DHPR, are also associated with a novel congenital muscular entity, mainly characterized by hypotonia, muscle weakness and severe wasting, elevated CK, narrow/cleft palate, ptosis, and potential cardiomyopathy (Fig. 4).

The two causative variants identified in the two families of this study were both located in exon 2 of *CACNB1*. As observed by long-read RNA sequencing in differentiated LHCN-M2 myotubes, the overwhelming majority of *CACNB1* transcript isoforms in these cells contain exon 2. Thus, loss-of-function variants in this exon can be interpreted as almost completely abolishing *CACNB1* function in skeletal muscle cells. This data was subsequently confirmed by the analysis of edited LHCN-M2 differentiated myotubes carrying a *CACNB1* truncating variant in exon 2. Nevertheless, it needs to be acknowledged that the transcriptomic profile, as well as the contractile and metabolic properties of LHCN-M2 myotubes, while similar, may not fully replicate the complexity of mature human skeletal muscle tissue, which represents a limitation of this study.

Previous studies in model organisms provide support for the pathogenicity of the variants we report in this article, as they clearly demonstrated the pivotal role of β1 in muscle function. *Cacnb1-*knockout mice were perinatal lethal due to respiratory failure, and at prenatal stages, β1-null embryos were described as having paralyzed muscles with morphological and histological features similar to those of the *Cacna1s* and *Ryr1* mutants [25]. Immunohistochemistry studies in myotubes from the *Cacnb1*-knockouts also revealed that β1 is required for targeting the α1S subunit to the cell membrane [25]. Analogously, zebrafish lines carrying recessive *cacnb1* nonsense mutations, so-called relaxed mutants, displayed similar paralysis. They also showed an immotile phenotype characterized by altered EC coupling and impaired α1S membrane localization [26].

In this work, we modeled one of the null variants identified in *CACNB1* (c.85-1G>A) in the human myoblast cell line LHCN-M2. Genetically modified cells carrying this variant were found with a severe reduction in the levels of the α1S protein when differentiating into myotubes, strongly suggesting that this is the pathomechanism underlying the condition of the patients of this study, or at least an important part of it. Under this hypothesis, the phenotypes associated with loss-of-function of either *CACNB1* or *CACNA1S* ought to be similar. Patients identified with recessive or dominant variants in *CACNA1S* that result in reduced or undetectable levels of α1S, were diagnosed with CM [29]. In general, these patients were described with antenatal/neonatal or early onset hypotonia, axial and generalized muscle weakness, and mild facial involvement including high arched palate and ophthalmoplegia. Variable degree of muscle atrophy and fibroadipose replacement was also noticed on MRI among these patients [29]. Overall, these features are comparable to those observed in the probands of the present report. However, CK levels seem to be an exception. While the three patients from the current study presented with elevated CK, only two of 11 patients, both from the same family, were reported with CK values higher than normal in the original report of the *CACNA1S*-related myopathy [29]. Similarly, other patients with *CACNA1S* pathogenic variants from subsequent publications have also been described with normal CK values [30]. In this context, it is important to take into account that β1 may have other functions than those associated with regulation of α1S levels, as suggested from its wide cellular distribution [19].

Although EC coupling abnormalities are mostly associated with CMs, these conditions are difficult to differentiate from congenital muscular dystrophies (CMDs) [31]. Both groups of disorders normally manifest since the neonatal period, have a number of features in common and are described with alterations in the structure and function of myofibers leading to muscle weakness. Clinical, and even genetic, overlap between CMDs and CMs has been observed. Indeed, pathogenic variants in *RYR1* and *SEPN1* can result in both CMs and CMD-like conditions [32, 33]. Of note, CMDs are characterized with elevated CK [34]. Hence, further histopathological evidence from additional patients would be required to classify the *CACNB1*-associated pathology into CM or CMD.

Moreover, as is the case for *CACNA1S* and *RYR1*, it cannot be ruled out that other kinds of *CACNB1* variants different than loss-of-function may lead to other types of muscular conditions, or even to conditions involving non-muscle tissues in which *CACNB1* is expressed. In this sense, a missense variant in *CACNB1* was suggested to be a contributing factor for malignant hyperthermia [35], and another amino acid change was observed in a patient with autism spectrum disorder (ASD) [36]. Rare tandem repeat expansions in *CACNB1* have also been linked with ASD [37].

In conclusion, *CACNB1* represents a novel congenital muscular disorder-associated gene, related to EC coupling. We propose adding this gene to CM/CMD panels, and suggest testing for *CACNB1* variants in patients manifesting severe muscle wasting with elevated CK and negative molecular analyses for previously known genes. Further cases with *CACNB1* disease-causing variants will help improve the understanding of the pathomechanism associated with this gene.

## Supplementary information

Supplemental Information

Supplemental Data 1

Supplemental Data 2

## Data Availability

Data supporting this article are included as Supplementary material. In addition, datasets and information not subjected to ethical restrictions are available from the corresponding authors upon reasonable request. Variants were submitted to ClinVar (accession numbers: SCV006302524 and SCV006303266).
