# Supplementary material for: N-terminal truncating variants in CACNB1 cause a new congenital muscular disorder
Source: Eur J Hum Genet. 2025 Sep 29;34(3):314–23. doi: 10.1038/s41431-025-01944-4 (PMC12963491; doi:10.1038/s41431-025-01944-4)
Supplement: Supplementary file 1 — Supplemental Information [file 41431_2025_1944_MOESM1_ESM.docx]

**SUPPLEMENTAL INFORMATION**

N-TERMINAL TRUNCATING VARIANTS IN *CACNB1* CAUSE A NEW CONGENITAL MUSCULAR DISORDER

Asier Iturrate, Nurit Assia Batzir, Ranit Jaron, David Garcia-Valentin, Julian Nevado, Jair Tenorio-Castano, Pablo Lapunzina, Kamila Lee, Rotem Greenberg, Dvora Sassi, Sharon Aharoni, Alla Kuzminsky, Lina Basel-Salmon, Naama Orenstein, Yakov Fellig, Shay Ben-Shachar, Dina Marek-Yagel, Victor L Ruiz-Perez

**TABLE OF CONTENTS**

1. **Supplemental materials and methods**
2. **Supplemental Figures 1-7**
3. **Supplemental Table 1**

**I. Supplemental materials and methods**

**Exome sequencing**

For family 1, DNA from peripheral blood was sequenced at Clalit Genomic Center (Petah Tikvah, Israel) on an Illumina NovaSeq 6000 platform, using the Illumina DNA prep with Enrichment (San Diego, CA, USA) with IDT xGen Exome Research Panel v2 kit. Resulting sequences were aligned against the human genome build GRCh37/UCSC hg19 and analyzed for sequence variants. Secondary analysis was performed with Illumina DRAGEN pipeline and tertiary analysis was carried out by the commercial Franklin by Genoox genetic analysis and variant classification tool (<https://franklin.genoox.com>). In the case of patient 3 (family 2), exome sequencing (ES) was conducted at NIMGenetics (Madrid, Spain). Exome libraries were generated from peripheral blood genomic DNA with SureSelectXT Human All Exon V6 (Agilent Technologies) and sequenced on a NovaSeq6000 platform. Reads were mapped to the GRCh37/hg19 genome reference and variants were annotated with DRAGEN v3.6.3 (Illumina).

**SNP-array hybridization**

Whole genome SNP-array hybridization was conducted by hybridizing peripheral blood genomic DNA to Illumina CytoSNP-850K BeadChip as described earlier (1). Homozygosity regions were assessed with GenomeStudio 2.0 software (Illumina).

**Cell culture**

The human myoblast cell line LHCN-M2 was acquired from Evercyte GmbH (Vienna, Austria). LHCN-M2 cells were seeded in 0.2% gelatin (Sigma-Aldrich) coated plates and cultivated in a humidified atmosphere at 37°C/5% CO2 in growth medium (GM): DMEM/M199 [4:1]; 20% fetal bovine serum; 10 ng/mL bFGF (R&D Systems); 1 μM dexamethasone (Sigma-Aldrich); and antibiotic-antimycotic (A-A) solution (Gibco, 100 units/mL penicillin, 100 μg/mL streptomycin, and 0.25 μg/mL amphotericin B). Differentiation of LHCN-M2 myoblasts into myotubes was achieved by switching the culture medium to myotube differentiation medium (DM) [DMEM/M199 (4:1); 2% horse serum; and A-A solution] and culturing the cells in this medium for 6 days. DM was replaced every two days. Cells were tested negative for mycoplasma.

**siRNA transfection**

LHCN-M2 myoblasts were seeded in GM at 5 x 10^5^/P60, 2.5 x 10^5^/P35, or 1 x10^5^/24-multiwell depending on experimental purposes. The next day, the culture medium was replaced with DM and cells were transfected with 50 nM of ON-TARGETplus *CACNB1* siRNA (SMARTpool, L-009359-00-0005, Dharmacon), or control siRNA (siNT) (ON-TARGETplus Non-targeting Pool, D-001810-10-05, Dharmacon), using DharmaFECT 1 reagent (Dharmacon). Forty-eight h post-transfection, DM was refreshed and cells were transfected again following the same procedure. Two days later, the culture medium was changed to new DM and at day 6 from the first transfection, the cultures were used for the corresponding experiments as indicated below.

**CRISPR-Cas9-based cytosine base editor (CBE) system**

The backbone of the CRISPR-Cas9-based cytosine base editor (CBE) construct PX459-CBEd was generated by exchanging the SpCas9 promoter and coding sequence of the pSpCas9(BB)-2A-Puro (PX459) V2.0 plasmid (Addgene, #62988) (ref. 2) with the TadA-CDd-SpCas9(D10A)-2xUGI cassette included in the SpCas9 TadCBEd vector (Addgene #193835) (ref. 3). IVA cloning methodology (4), Platinum™ SuperFi II High Fidelity polymerase (ThermoFisher) and primers listed in Supplemental Table 1 were used in this procedure. Additionally, a V106W substitution was introduced into the sequence of the TadA-derived CBE using site directed mutagenesis to generate the PX459-CBEd(V106W) plasmid (3). Finally, a sgRNA targeting the acceptor splice site of exon 2 of *CACNB1,* 5’-TGCCCTGAAGAGGCCAGGAA-3’, was cloned into the PX459-CBEd(V106W) construct using *BbsI* restriction sites. The DNA sequence of the final construct was verified by Whole Plasmid Sequencing using Oxford Nanopore technology provided by Eurofins Genomics.

**LHCN-M2 genome editing**

LHCN-M2 human myoblasts (2.5 x 10^5^/P60) were plated and next day transfected with 5.5 μg of the PX459-CBEd(V106W) construct containing the sgRNA against *CACNB1* indicated above using Lipofectamine3000 (ThermoFisher). Twenty-four h after transfection, the medium was replaced with fresh GM and cells were left to recover for another day before undergoing puromycin selection for 72 h (2 μg/mL in GM). Transfected cells were cultured for one additional week and seeded at approximately 50 cells/P100. When colonies reached around 100 cells, they were picked up and transferred to 24-multiwell plates to establish monoclonal cell lines. Genomic DNA from individual clonal cell lines was extracted with NucleoSpin DNA RapidLyse kit (Macherey-Nagel) and used to amplify a DNA fragment comprising *CACNB1* exon 2 and flanking regions (Supplemental Table 1). The c.85-1G>A nucleotide change introduces a new restriction site for *EcoNI* and PCR products were screened by restriction digestion with this enzyme. Homozygous cell lines for the desired mutation were selected and their genotype confirmed by Sanger Sequencing.

**RT-PCR and RT-qPCR**

Patient peripheral blood was collected in Tempus™ Blood RNA Tubes (ThermoFisher) and RNA extracted with Tempus™ Spin RNA Isolation Kit (ThermoFisher). Whole blood control RNA was obtained from Takara. Synthesis of cDNA from blood tissue was carried out with the SuperScript IV First-Strand Synthesis System kit (ThermoFisher), using 500 ng of total RNA and random hexamers (Promega). RT-PCR was performed with 25 ng of cDNA (assuming 1:1 RNA to cDNA conversion) and primers listed in Supplemental Table 1. For myotube studies, 2.5 x 10^5^ LHCN-M2 cells were seeded in GM in P35 dishes and subsequently differentiated as indicated above. RNA was extracted at day 6 of myotube differentiation with the Speedtools Total RNA Extraction Kit (Biotools). In the case of undifferentiated myoblasts, LHCN-M2 cells were plated at 1 x 10^5^/P35 density in GM and 48 h later RNA was isolated as for myotubes. Reverse transcription in samples from cultured cells was achieved with the high-capacity reverse-transcription kit from Applied Biosystem, using 250 ng of total RNA and random primers. RT-PCR was performed with 15 ng of cDNA and oligonucleotides indicated in Supplemental Table 1. Amplified products were treated with ExoSAP-IT (Applied Biosystem) and Sanger sequenced. RT-qPCR experiments were conducted with TaqMan real-time PCR gene expression assays (Applied Biosystems) in a QS7 Flex Real-Time PCR system (Applied Biosystem) using 2.5 ng of cDNA and a final reaction volume of 10 μL. Each sample was analyzed in triplicate. The following TaqMan gene expression assays were used: *CACNB1* (exon 7a containing isoforms) Hs01120681_m1; *CACNB1* (all isoforms) Hs00609497_m1; *CACNA1S* Hs00163885_m1; *GAPDH* Hs99999905_m1; and *TBP* Hs00427621_m1. Relative quantification of each transcript was determined with the 2^−ΔΔCt method. Ct values of target genes were normalized against the geometric mean of the Ct values of housekeeping genes *GAPDH* and *TBP*. To calculate ΔΔCt, the ΔCt mean value from three independent biological replicates of the control sample (wild-type LHCN-M2 myotubes) was used as calibrator.

**Long-read RNA sequencing**

Long-read whole transcriptome RNA sequencing (Iso-Seq) of 6-days differentiated wild-type LHCN-M2 myotubes was provided by Novogene Europe. Briefly, 300 ng of total RNA (RIN>8) were used for library preparation using the Kinnex full-length RNA kit (PacBio) and sequenced with a PacBio Revio sequencer. High-Fidelity reads were processed using the SMRT Link software to obtain full-length non-chimeric (FLNC) reads. For novel isoform discovery, redundant FLNC reads corresponding to the same transcript were clustered using the hierarchical n*log(n) algorithm to generate consensus sequences. These consensus reads were then aligned using minimap2, and novel isoforms were identified with Pigeon software, based on the reference genome (GRCh38) and annotations from Ensembl (release 82). For isoform quantification, all FLNC reads were aligned and then quantified with IsoQuant, using annotations from Ensembl (release 82) along with novel transcript isoforms obtained from the isoform discovery step. Alignment and annotation files were visualized with IGV 2.15.

**Immunoblotting**

LHCN-M2 cells were seeded at 5 x 10^5^ cells/P60 in GM and 24h later changed to DM. After 6 days of differentiation, myotubes were lysed in RIPA buffer (150 mM NaCl, 50 mM Tris-HCl, 2 mM EDTA, 0.5% sodium deoxycholate, 0.1% SDS, 1% NP-40) supplemented with a protease inhibitor cocktail (Sigma-Aldrich; P8340), phosphatase inhibitors (Sigma-Aldrich, P0044 and P5726) and 1 mM PMSF. Protein extracts from undifferentiated LHCN-M2 cells (2 x 10^5^/P60) were obtained from cultures maintained 48 h in GM. BCA colorimetric assay (ThermoFisher) was carried out for protein quantification. Samples were boiled in Laemmli buffer at 95ºC for 5 min except for α1S analysis that were heated for 30 min at 37ºC before loading into SDS-PAGE gels. Proteins were blotted onto nitrocellulose membranes (Amersham) and used for Western Blot analysis following standard procedures. Primary antibodies: anti-Cavβ1 (NeuroMab #75-052; 1:1,000), anti-Cavα1s (Sigma-Aldrich #HPA048892; 1:1,000), anti-MyHC (R&D Systems #MAB4470; 1:10,000), anti-tubulin (Sigma-Aldrich #T9026; 1:240,000) and anti-vinculin (Cell Signaling #13901; 1:20,000). HRP-conjugated secondary antibodies (Jackson ImmunoResearch; 1:10,000) were incubated for 1 hour at RT. Immunoblot membranes were developed with enhanced chemiluminescence (ECL) reagent (Cytiva) and exposed to Agfa X-ray films. Densitometry analysis of α1S was carried out using ImageJ/Fiji software. For each sample the intensity of the α1S signal referred to tubulin (α1S/tubulin) was normalized against the levels of the differentiation marker MyHC referred to vinculin (MyHC/vinculin) measured in the same sample. In each biological replicate, α1S normalized values were referred to the mean value of two control samples (untreated and siNT treated differentiated myotubes).

**Immunofluorescence**

LHCN-M2 myoblasts (1 x 10^5^ cells/well (24-multiwell plate)) were plated on gelatin-covered coverslips in GM and transferred to DM on the next day. After 6 days of culture in DM, myotubes were fixed with 4% PFA/PBS for 15 min at RT, permeabilized for 15 min in 0.1% Triton X-100/PBS and blocked for one h in blocking buffer (4% goat serum + 1% BSA in 0.05% Triton X-100/PBS). Primary antibodies diluted in blocking buffer were incubated overnight at 4ºC in a wet chamber. Primary antibodies: anti-Cavβ1 (NeuroMab #75-052; 1:500), anti-Cavα1s (Sigma-Aldrich #HPA048892; 1:500), anti-RYR1 (abcam #ab2868; 1:100) and anti-MyHC (R&D Systems #MAB4470; 1:5,000). Coverslips were subsequently washed with PBS and 0.05% Tween20/PBS (PBS-T), and fluorescence-conjugated species- and isotype-specific secondary antibodies (ThermoFisher; 1:1,000) were added for 1 h at RT. Following additional PBS/PBS-T washes, nuclei were stained with DAPI for 5 min (ThermoFisher; #D1306; 1:2,000). Coverslips were mounted using Prolong Diamond (ThermoFisher) and images acquired in a STELLARIS 8 STED spectral confocal microscope (Leica Microsystems). Images were processed using Fiji/ImageJ software.

**Statistical analysis**

Statistical analysis was performed using GraphPad Prism 10. Normal distribution of data was confirmed using Shapiro-Wilk test or the Skewness normality score. Differences among samples were evaluated with one-way analysis of variance (ANOVA) test followed by Dunnett’s post-hoc multiple comparisons test.

**References**

1. Palencia-Campos A, Ullah A, Nevado J, Yildirim R, Unal E, Ciorraga M, et al. GLI1 inactivation is associated with developmental phenotypes overlapping with Ellis-van Creveld syndrome. Hum Mol Genet. 2017; 26:4556-71.

2. Ran FA, Hsu PD, Wright J, Agarwala V, Scott DA, Zhang F. Genome engineering using the CRISPR-Cas9 system. Nat Protoc. 2013; 8:2281-308.

3. Neugebauer ME, Hsu A, Arbab M, Krasnow NA, McElroy AN, Pandey S, et al. Evolution of an adenine base editor into a small, efficient cytosine base editor with low off-target activity. Nat Biotechnol. 2023; 41:673-85.

4. Garcia-Nafria J, Watson JF, Greger IH. IVA cloning: A single-tube universal cloning system exploiting bacterial In Vivo Assembly. Sci Rep. 2016; 6:27459.

**II. Supplemental Figures**

**
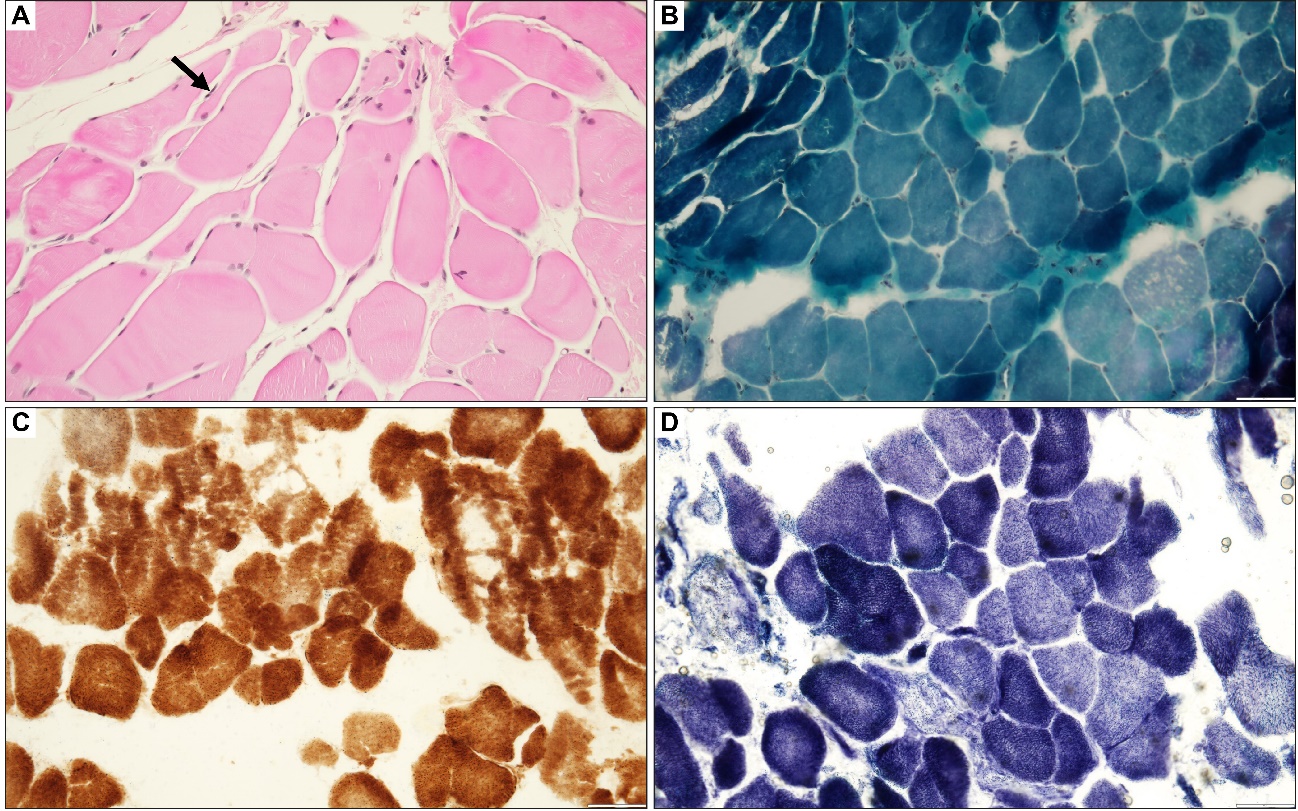
**

**Supplemental Figure 1. Histopathological analysis of a skeletal muscle biopsy from P2. A.** Paraffin embedded sections of skeletal muscle tissue stained with hematoxylin-eosin showing no significant alterations, except for few atrophic angulated myofibers (arrow). **B-D**. Frozen sections stained with Modified Gomori-Trichrome (B), SDH/COX (C) and NADH (D) showing no remarkable findings. No ragged-red fibers, rods or cores are seen. COX is not reduced. Scale bars: 50 µm.

**
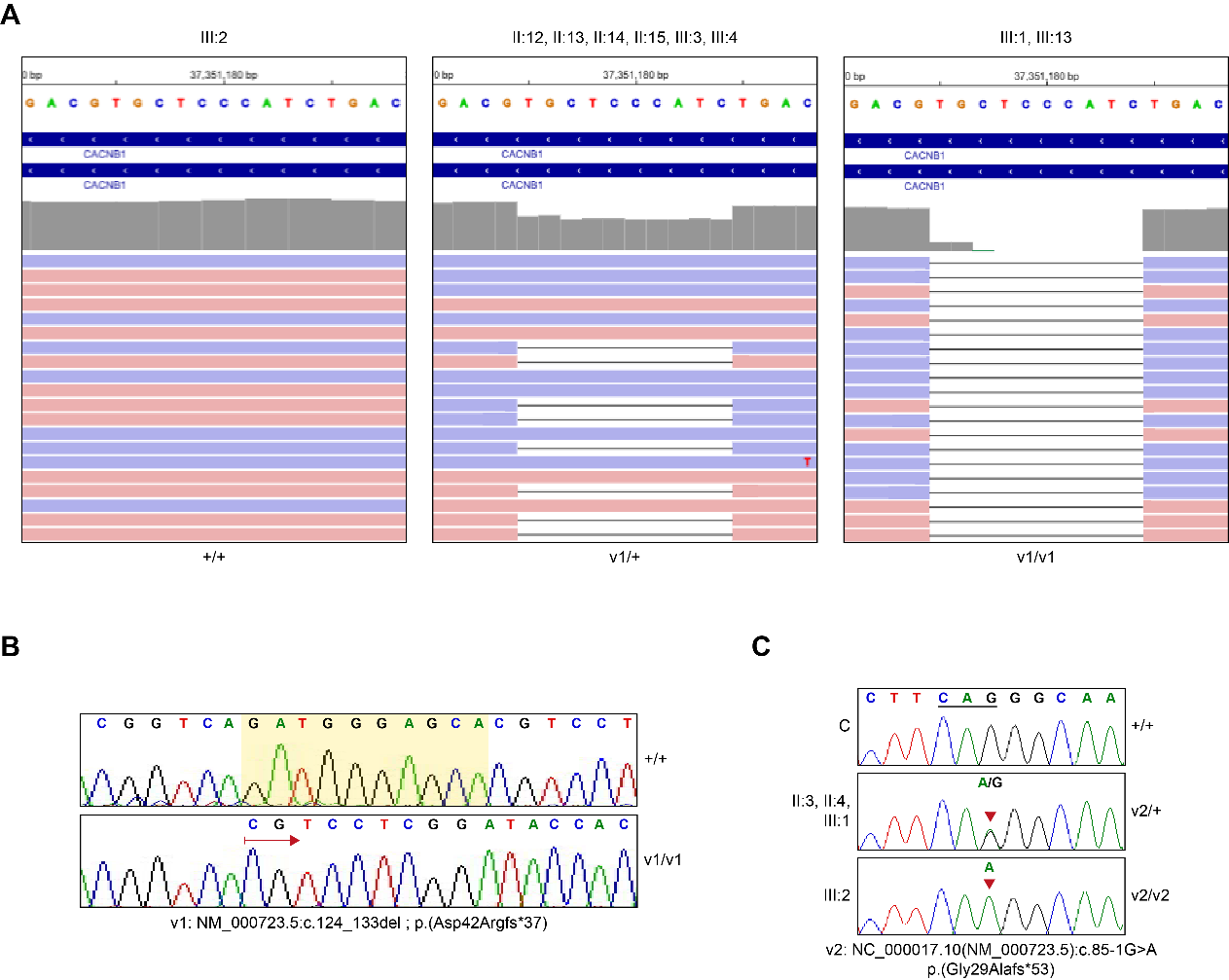
**

**Supplemental Figure 2. Family segregation and Sanger sequencing validation of *CACNB1* variants. A.** Representative IGV visualizations of paired-end sequencing reads corresponding to a region of *CACNB1* exon 2 of probands (v1/v1), heterozygotes (v1/+), and an individual with wild-type sequence (+/+) of family 1. **B**. Genomic DNA Sanger sequencing chromatograms of *CACNB1* exon 2 from a proband (v1/v1) and a healthy individual with normal sequence (+/+) of family 1. Deleted nucleotides in the proband are colored in yellow in the control sequence. The arrow marks the position in which the nucleotide sequence of the control and the proband begin to differ due to the homozygous deletion. **C**. Sanger Sequencing chromatograms of *CACNB1* exon 2 from genomic DNA of the proband of family 2 (v2/v2), and a control individual (C; +/+). A representative chromatogram for the unaffected parents and brother of P3 is also included (v2/+). Nucleotides of the acceptor splice site of *CACNB1* exon 2 are underlined in the control. Arrowheads mark the position of variant 2.

**
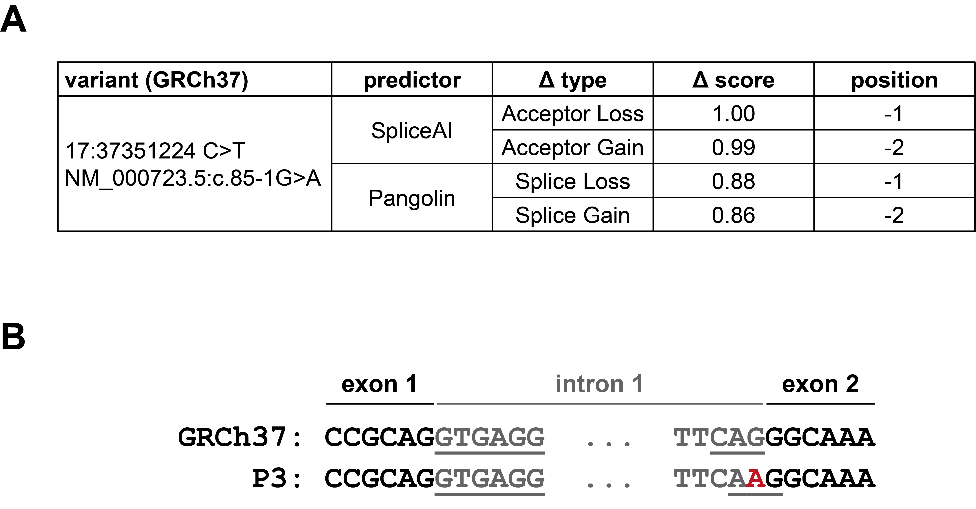
**

**Supplemental Figure 3. Bioinformatic analysis of *CACNB1* c.85-1G>A variant. A.** SpliceAI and Pangolin splicing prediction scores for the c.85-1G>A variant (https://spliceailookup.broadinstitute.org/). **B.** Nucleotide sequences of exon 1 donor and exon 2 acceptor splice sites from the reference human genome assembly (GRCh37) and the proband of family 2 (P3). Splice site sequences are underlined and the c.85-1G>A variant is depicted in red.

**
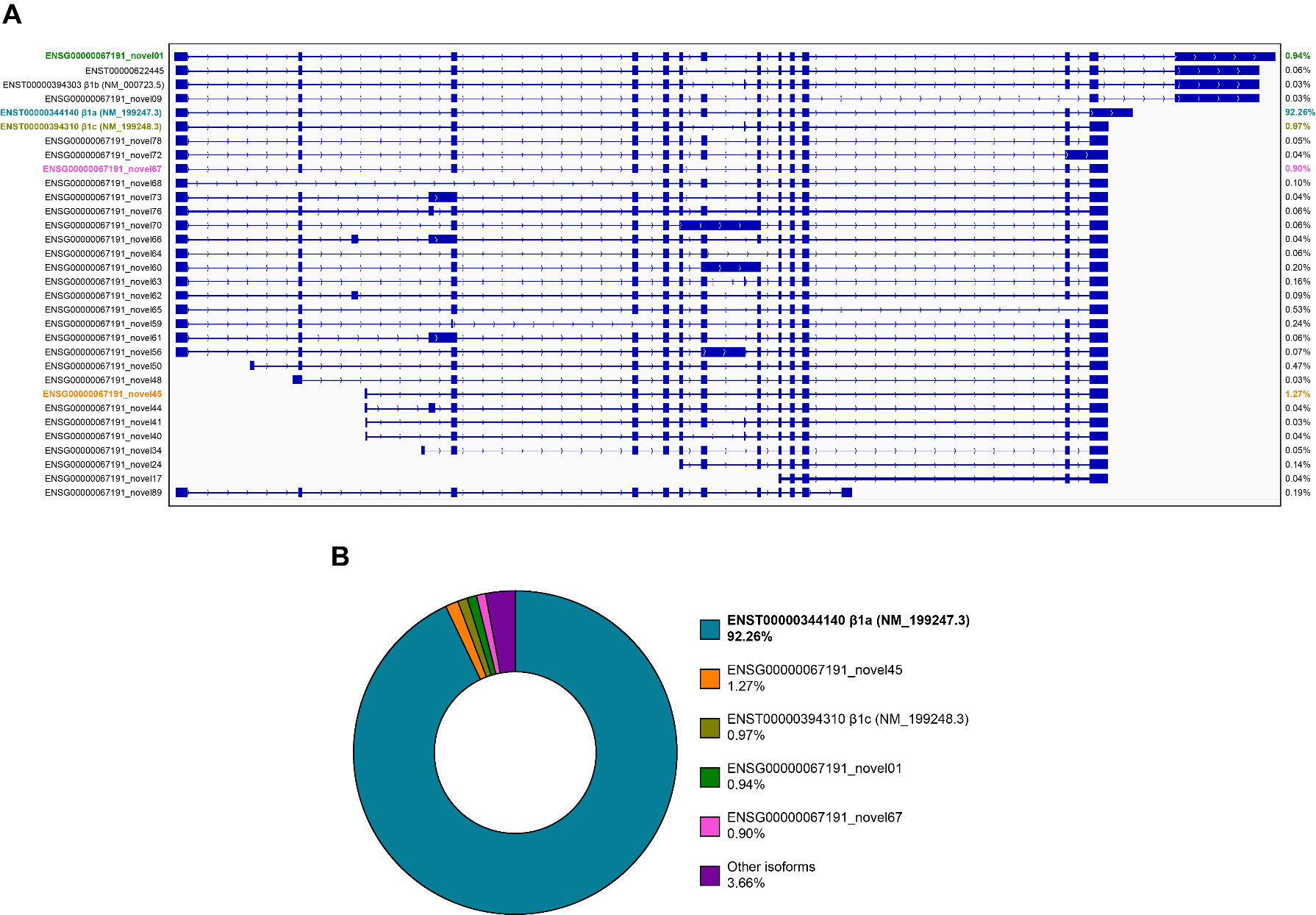
**

**Supplemental Figure 4.** **Repertoire of *CACNB1* transcript isoforms in LHCN-M2 myotubes identified by long-read RNA sequencing. A.** IGV snapshot of *CACNB1* transcript isoforms identified in LHCN-M2 myotubes with at least 5 reads according to IsoQuant quantification (32 isoforms are shown). Novel isoforms and isoforms annotated in Ensembl (release 82) are indicated. Read percentages aligning to each isoform are shown on the right. The 5 most abundant transcript isoforms are colored. **B.** Pie chart showing read percentages for the top 5 most abundant *CACNB1* transcript isoforms in LHCN-M2 myotubes. The percentage of reads corresponding to the remaining transcripts is also indicated.

**
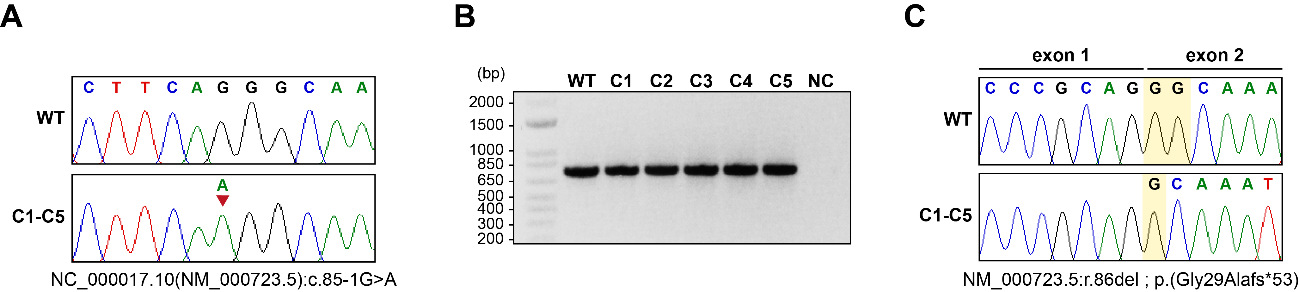
**

**Supplemental Figure 5. Genetic characterization of LHCN-M2 clonal cell lines harboring the c.85-1G>A *CACNB1* variant. A**. Representative genomic DNA electropherograms of LHCN-M2 parental (WT) and *CACNB1*-edited clonal cell lines (C1-C5) homozygous for the c.85-1G>A variant (read arrowhead). **B**. Agarose gel image showing the result of an RT-PCR experiment between exons 1 and 7a of *CACNB1* performed in myotubes from parental LHCN-M2 cells (WT) and edited clonal cell lines (C1-C5), n=3. NC: non-template control. **C.** Sanger sequencing chromatograms of WT and mutant (C1-5) RT-PCR products shown in panel B demonstrating deletion of the first nucleotide of exon 2 in the edited cells. Nucleotides highlighted in yellow illustrate the deletion.

**
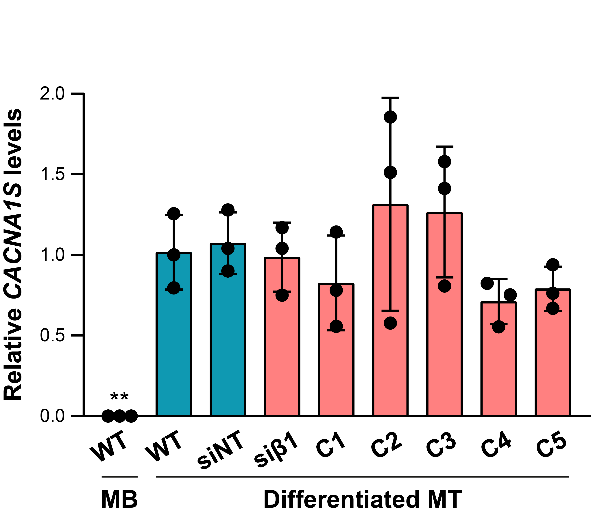
**

**Supplemental Figure 6. *CACNA1S*** **mRNA levels in siRNA-treated and *CACNB1*-edited LHCN-M2 cells**. Relative quantification of the levels of *CACNA1S* mRNA by RT-qPCR in control (WT) and c.85-1G>A *CACNB1*-edited (C1-C5) LHCN-M2 differentiated myotubes (MT) using TaqMan gene expression assays. LHCN-M2 myoblasts (MB), as well as myotubes treated with siRNA against all *CACNB1* transcript isoforms (siβ1), or with a non-targeted siRNA (siNT), were included in this experiment. Data are expressed as mean ± SD (n=3). ∗∗p < 0.01. One-way ANOVA with Dunnett’s multiple comparisons test relative to the WT-MT control sample. *CACNA1S* expression levels were normalized to the geometric mean of *GAPDH* and *TBP* values. The ΔCt mean value of wild-type (WT) differentiated myotubes was used as the calibrator sample.

**
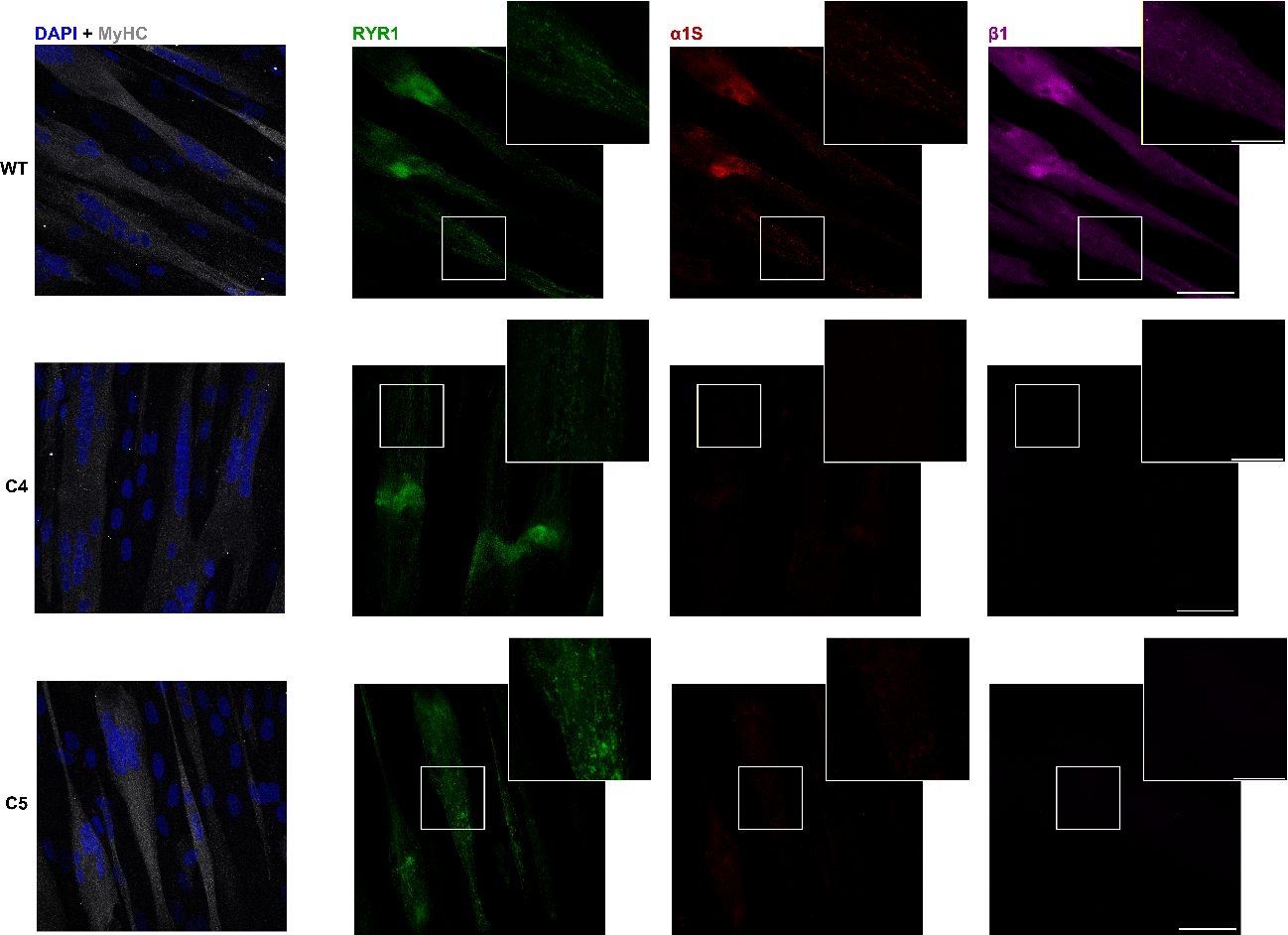
**

**Supplemental Figure 7. Immunofluorescence analysis of c.85-1G>A *CACNB1*-edited LHCN-M2 clonal cells.** Maximum intensity Z-projection of confocal immunofluorescence images of control (WT) and c.85-1G>A edited (C4, C5) LHCN-M2 myotubes stained for MyHC, RYR1, α1S and β1. n=2. Nuclei are stained in blue with DAPI in the MyHC images. Boxed areas are digitally magnified in inset images. Scale bars: 50 µm (overview images), and 20 µm (magnified insets).

**III. Supplemental Table 1.** **Primers used in this work**

| **Primer** | **Sequence (5' - 3')** | **Use** |
| --- | --- | --- |
| PX459 IVA Fw | CCAAGAAGAAGAGGAAAGTCGAATTCGGCAGTGGAGAGGG | PX459-CBEd IVA cloning |
| PX459 IVA Rv | TAATAACTAGTCAATAATCAATGTCGGTACCTCTAGAGCCATTTG | PX459-CBEd IVA cloning |
| TadCBEd IVA Fw | GACATTGATTATTGACTAGTTATTA | PX459-CBEd IVA cloning |
| TadCBEd IVA Rv | GACTTTCCTCTTCTTCTTGG | PX459-CBEd IVA cloning |
| PX459-CBEd(V106W) Fw | CGCGTGGTGTTTGGCTGGAGGAACTCAAAAAGAGGCG | V106W mutagenesis |
| PX459-CBEd(V106W) Rv | GCCAAACACCACGCGGCCGA | V106W mutagenesis |
| *CACNB1* intron1 Fw | CTCTCCGTGAGGATGGGAGA | *CACNB1* c.85-1G>A genotyping |
| *CACNB1* intron2 Rv | GGCTGTCCCTGAATGAGATGA | *CACNB1* c.85-1G>A genotyping |
| *CACNB1* exon1 Fw | CTCCTCTCCATGGTCCAGAA | RT-PCR of *CACNB1* in blood and in LHCN-M2 |
| *CACNB1* exon9 Rv | TAACCTCGTAGCCCTTGAGC | RT-PCR of *CACNB1* in blood |
| *CACNB1* exon7A Rv | GGGGTGGTGACACTGCTAAC | RT-PCR of *CACNB1* in LHCN-M2 |
